# Acidification effects on isolation of extracellular vesicles from bovine milk

**DOI:** 10.1371/journal.pone.0222613

**Published:** 2019-09-16

**Authors:** Md. Matiur Rahman, Kaori Shimizu, Marika Yamauchi, Hiroshi Takase, Shinya Ugawa, Ayaka Okada, Yasuo Inoshima

**Affiliations:** 1 The United Graduate School of Veterinary Sciences, Gifu University, Gifu, Gifu, Japan; 2 Laboratory of Food and Environmental Hygiene, Cooperative Department of Veterinary Medicine, Gifu University, Gifu, Gifu, Japan; 3 Department of Medicine, Sylhet Agricultural University, Sylhet, Bangladesh; 4 Core Laboratory, Graduate School of Medical Sciences, Nagoya City University, Mizuho-cho, Mizuho-ku, Nagoya, Aichi, Japan; 5 Department of Anatomy and Neuroscience, Graduate School of Medical Sciences, Nagoya City University, Mizuho-cho, Mizuho-ku, Nagoya, Aichi, Japan; 6 Education and Research Center for Food Animal Health, Gifu University (GeFAH), Gifu, Gifu, Japan; 7 Joint Graduate School of Veterinary Sciences, Gifu University, Gifu, Gifu, Japan; University of Cincinnati College of Medicine, UNITED STATES

## Abstract

Bovine milk extracellular vesicles (EVs) attract research interest as carriers of biologically active cargo including miRNA from donor to recipient cells to facilitate intercellular communication. Since toxicity of edible milk seems to be negligible, milk EVs are applicable to use for therapeutics in human medicine. Casein separation is an important step in obtaining pure EVs from milk, and recent studies reported that adding hydrochloric acid (HCl) and acetic acid (AA) to milk accelerates casein aggregation and precipitation to facilitate EV isolation and purification; however, the effects of acidification on EVs remain unclear. In this study, we evaluated the acidification effects on milk-derived EVs with that by standard ultracentrifugation (UC). We separated casein from milk by either UC method or treatment with HCl or AA, followed by evaluation of EVs in milk serum (whey) by transmission electron microcopy (TEM), spectrophotometry, and tunable resistive pulse sensing analysis to determine EVs morphology, protein concentration, and EVs size and concentration, respectively. Moreover, we used anti-CD9, -CD63, -CD81, -MFG-E8, -HSP70, and -Alix antibodies for the detection of EVs surface and internal marker proteins by western blot (WB). Morphological features of EVs were spherical shape and similar structure was observed in isolated EVs by TEM. However, some of the EVs isolated by HCl and AA had shown rough surface. Although protein concentration was higher in whey obtained by UC, EV concentration was significantly higher in whey following acid treatment. Moreover, although all of the targeted EVs-marker-proteins were detected by WB, HCl- or AA-treatments partially degraded CD9 and CD81. These findings indicated that acid treatment successfully separated casein from milk to allow efficient EV isolation and purification but resulted in partial degradation of EV-surface proteins. Our results suggest that following acid treatment, appropriate EV-surface-marker antibodies should be used for accurate assess the obtained EVs for downstream applications. This study describes the acidification effects on EVs isolated from bovine milk for the first time.

## Introduction

Extracellular vesicles (EVs) are membranous nanoparticles ranging in size from 30 nm to 150 nm and secreted from living cells [1]. EVs can be found in most bodily fluids, including blood, breast milk, urine, saliva, malignant ascites, amniotic fluid, and tears [2,3]. Because EVs contain miRNA, mRNA, DNA, lipids, and proteins [4], they represent vehicles for delivery of biologically active cargo from donor to recipient cells to facilitate intercellular communication and the exchange of genetic information [4,5]. Recently, EVs were identified as promising tools for cancer therapy in human medicine [6]. Ascite-derived EVs were successfully used as an alternative choice for immunotherapy of advanced colorectal cancer [7], and Ohno et al. [8] reported the successful EV-mediated delivery of anti-tumor miRNA to breast cancer cells *in vitro* and *in vivo*. These data suggest EVs as natural carriers of miRNA and potentially useful for drug-delivery systems and inducers of signaling during inflammation and infection [9,10]. Moreover, EVs represent potential biomarkers for cancer diagnosis, as glioblastoma-derived EVs contain *epithelial growth factor receptor vIII* mRNA as a marker of tumor formation, progression, and response to therapy [11]. Furthermore, miR-21 in serum EVs is reportedly a potential biomarker for hepatocellular carcinoma [12].

EVs isolated from bovine milk [13] contain mRNA associated with major milk proteins, as well as immune-related miRNAs, such as caseins, β-lactoglobulin, miR-101, and miR-150 [14,15]. Additionally, milk-derived EVs play an important role in infant growth [16] and immune-system development [17] in mammals, indicating that milk EVs facilitate intercellular communication. Moreover, milk-derived EVs provide novel information concerning biomarkers potentially helpful for dairy herd management, including the physiological state of the animal [13], its metabolic condition [18], and pathogen infections [19,20].

Bovine milk contains other colloidal structures with milk EVs, such as milk-fat globules (MFGs) and casein micelles [21,22]. Casein is the major milk protein and comprises >80% of the total protein in milk in contrast to 35% in human breast milk [23]. This large amount of casein in milk increases the difficulty of EV isolation and purification. Numerous reports have described methods for EV isolation and purification from milk, with most involving centrifugation, ultracentrifugation (UC), sucrose-density gradients, fast protein liquid chromatography, gel filtration, and/or commercial EV-isolation kits [24–26]. However, all of these methods are time-consuming and require multiple steps to remove other non-EV proteins. Recent studies described the positive effect of low pH on EV yield and purity [27], and that adding acetic acid (AA) promoted casein removal during EV isolation [28]. We previously revealed isoelectric precipitation of caseins by hydrochloric acid (HCl) treatment as efficacious for removing casein, with this method also reducing operation time; however, isolated milk EVs showed a rough surface [29], indicating that acidification might have affected EV-surface-marker proteins during isolation. In the present study, we evaluated the effects of acidification on EV isolation and purification from milk, and revealed partial degradation of EV-surface-marker proteins. This is the first study reporting about acidification effects on EVs.

## Materials and methods

### Bovine milk samples

Milk samples were collected from healthy dairy cows at the Field Science Center, Yanagido Farm, Gifu University (Gifu, Japan). Milk was transported from the farm to the laboratory within 10 min after collection, with a cooling box full of ice used to prevent protein deterioration in the milk during transport. This study was approved by the Gifu University Animal Care and Use Committee (No. 17046).

### EV isolation

Fresh milk (~1,000 mL) was centrifuged at 2,000×*g* at 4°C for 20 min using an A508-C rotor (Kubota, Tokyo, Japan) in a model 7000 centrifuge (Kubota) to remove MFGs, somatic cells, debris, and the cream layer [29]. To evaluate the effects of acidification on EV isolation, three methods were compared as follows.

### UC

Defatted milk (500 mL) was subjected to UC using a P42A angle rotor (Hitachi Koki, Tokyo, Japan) in a Himac CP80WX ultracentrifuge (Hitachi Koki) at 12,000×*g* for 1 h at 4°C, after which the middle layer was collected, and the lower slush portion along with the pellet was discarded. This fraction was again subjected to UC at 35,000×*g* for 1 h, after which the middle layer was collected and subjected to another round of UC at 75,000×*g* for 3 h at 4°C. The supernatant was collected and filtered using 1.0-, 0.45-, and 0.2-μm filters (GA-100, C045A0474, and C020A047A, respectively; Advantec, Tokyo, Japan) to remove remaining MFGs and debris and obtain milk serum (whey).

### HCl treatment

Defatted milk (250 mL) was added to an equal volume of distilled water (DW), and after pre-warming at 37°C for 10 min, the pH was adjusted to 4.6 with 6N HCl (Wako, Osaka, Japan) to promote casein aggregation [30]. Casein was separated by centrifugation at 5,000×*g* at 25°C for 20 min using an R14A rotor (Hitachi Koki) in a Himac CR20GII centrifuge (Hitachi Koki). The supernatant was filtered as described above in order to obtain whey.

### AA treatment

Defatted milk (250 mL) was pre-warmed at 37°C for 10 min and mixed with AA [milk/AA = 100% (vol/vol)] and stirred for 5 min at room temperature (RT), followed by centrifugation at 5,000×*g* at 25°C for 20 min using an R14A rotor (Hitachi Koki) in a Himac CR20GII centrifuge (Hitachi Koki). Casein was pelleted, and the supernatant was filtered as described above in order to obtain whey.

### Transmission electron microscopy

EVs morphology were observed by transmission electron microscopy (TEM) as described previously [29] with slight modifications. EVs pellet solutions were applied into glow-discharged carbon support films on copper grids and stained with 2% uranyl acetate in DW, following examined by an electron microscope, JEM-1400 Plus (JEOL, Tokyo, Japan) at 100 kV.

### Protein concentration

Protein concentration of the recovered whey was measured using Lowry’s method [30] using a DC protein assay kit (500–0113, 500–0114, 500–0115, and 5000007; Bio-Rad Laboratories, Hercules, CA, USA) by a spectrophotometry, GeneQuant100 (GE Healthcare, Chicago, IL, USA).

### Tunable resistive pulse sensing analysis

**Tunable resistive pulse sensing (**TRPS) analysis was performed according to the manufacturer’s instructions in order to measure EV size and concentration using a qEV single and qNano TRPS instrument (Izon Science, Christchurch, New Zealand).

### Western blot analysis

Whey was obtained by UC method or HCl- or AA-treatment, and protein concentration was adjusted to 5μg/μL with distilled water. A 20% volume of 6×sodium dodecyl sulfate (SDS) sample buffer [0.05M Tris-HCl (pH 6.8), 6% 2-mercaptoethanol, 2% SDS, 5% glycerol, and bromophenol blue) was added to the whey, which was then boiled at 95°C for 5 min and centrifuged, followed by loading of the sample on to an SDS-polyacrylamide gel. The gel containing the proteins was transferred to an Immobilon-P polyvinylidene difluoride membrane (Merck Millipore, Cork, Ireland) and blocked with 5% ovalbumin or 5% non-fat dry milk in Tris-buffered saline [0.1M Tris-HCl (pH 8.0) and 0.03M NaCl] containing 0.1% Tween-20 (TBST) at room temperature (RT) for 30 min. The membrane was then incubated with primary antibodies (Table 1) diluted with 1% ovalbumin or 1% non-fat dry milk in TBST at RT for 1 h, followed by washing with TBST three times.

**Table 1:** Primary antibodies used for detection of EVs-marker proteins by western blot.

Antibody	Identity	Molecular size (kDa)	Origin	Ab nature	Supplier*	Product number
CD9	IVA50	24	Mouse	Monoclonal	NB	NB500-494
CD63	M-13	60	Goat	Polyclonal	SCB	SC-31214
CD63	T-14	63	Goat	Polyclonal	SCB	SC-25183
CD81	B-11	24	Mouse	Monoclonal	SCB	SC-166029
CD81	12C4	24	Mouse	Monoclonal	CB	SHI-EXO-MO3
MFG-E8	6F11	53, 57	Mouse	Monoclonal	NU	A kind gift
MFG-E8	M-135	57	Rabbit	Polyclonal	SCB	SC-33546
HSP70	N27F3-4	95	Mouse	Monoclonal	ELS	ADI-SPA-820 F
Alix	3A9	95	Mouse	Monoclonal	SC	SC-53538
ApoA-1	B-10	28	Mouse	Monoclonal	SC	SC-58230
ApoA-1	069–01	28	Mouse	Monoclonal	SC	SC-376818

For membrane blocking in WB, 5% ovalbumin (CD9 and CD81 antibodies) and 5% non-fat dried milk (CD63, MFG-E8, HSP70, Alix, and ApoA1 antibodies) in TBST were used.

* NB, Novus Biologicals; SCB, Santa Cruz Biotechnology; CB, Cosmo Bio; NU, Nagoya University; ELS, Enzo Life Science.

The secondary antibodies, either an anti-mouse IgG sheep antibody conjugated with horseradish peroxidase (HRP; 1: 2,000; NA9310; GE Healthcare, Little Chalfont, UK) or an anti-goat IgG donkey antibody conjugated with HRP (1: 2,000; sc-3851; Santa Cruz Biotechnology, Dallas, TX, USA) were diluted in TBST at RT for 1 h, followed by membrane washing with TBST three times. Peroxidase activity was detected using a Pierce ECL Plus western blotting substrate (Thermo Fisher Scientific, Waltham, MA, USA) and visualized using a ChemiDoc XRS+ (Bio-Rad Laboratories) or an LAS 4000 mini system (Fujifilm, Tokyo, Japan).

### Statistical analysis

Data were obtained from three independent experiments and expressed as the mean ± standard deviation by one-way ANOVA followed by Kruskal Wallis H-test (Post hoc test). Statistical significance was determined at *p*<0.05.

## Results

### TEM

**Fig 1:**
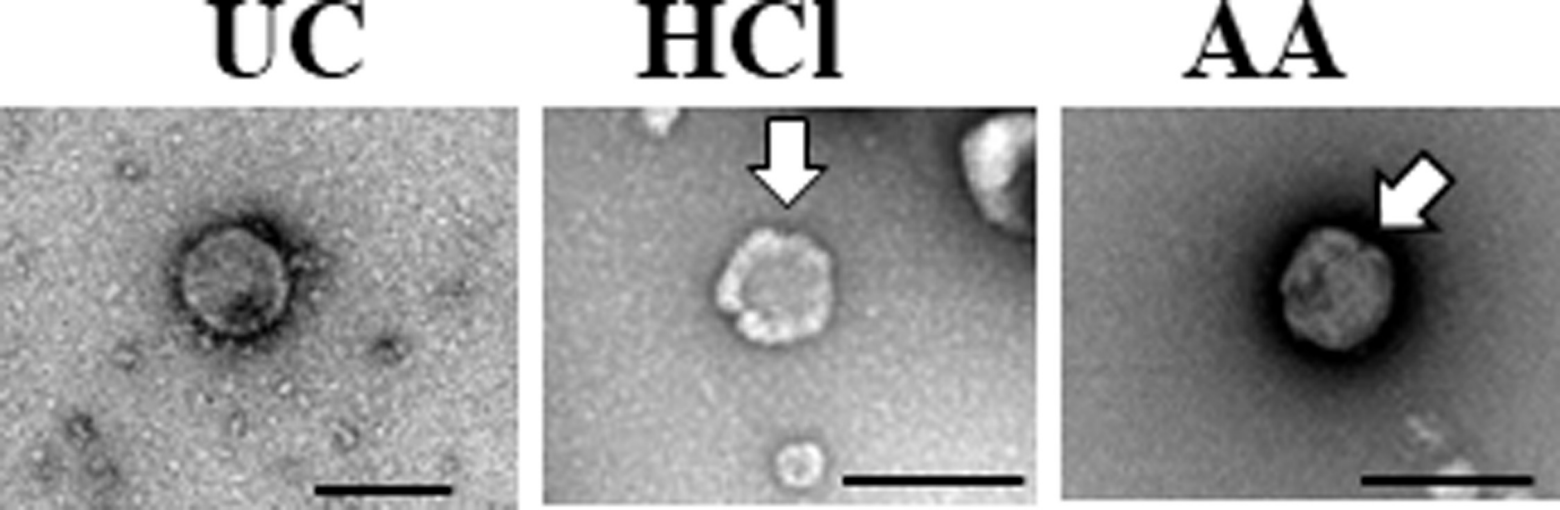
Transmission electron microscopy (TEM) observation of bovine milk EVs. Similar spherical shape morphology was observed in EVs isolated by UC method, HCl-, or AA-treatments by TEM. However, rough surfaces (arrows) were observed in some of the EVs isolated by HCl- or AA-treatments. Scale bar showed 200 nm.

Using the TEM, the morphological features of EVs isolated by UC method, HCl- and AA-treatments showed a similar spherical shape (Fig 1). However, rough surface was observed some of the EVs isolated by using HCl and AA (Fig 1).

### Protein concentration

**Fig 2:**
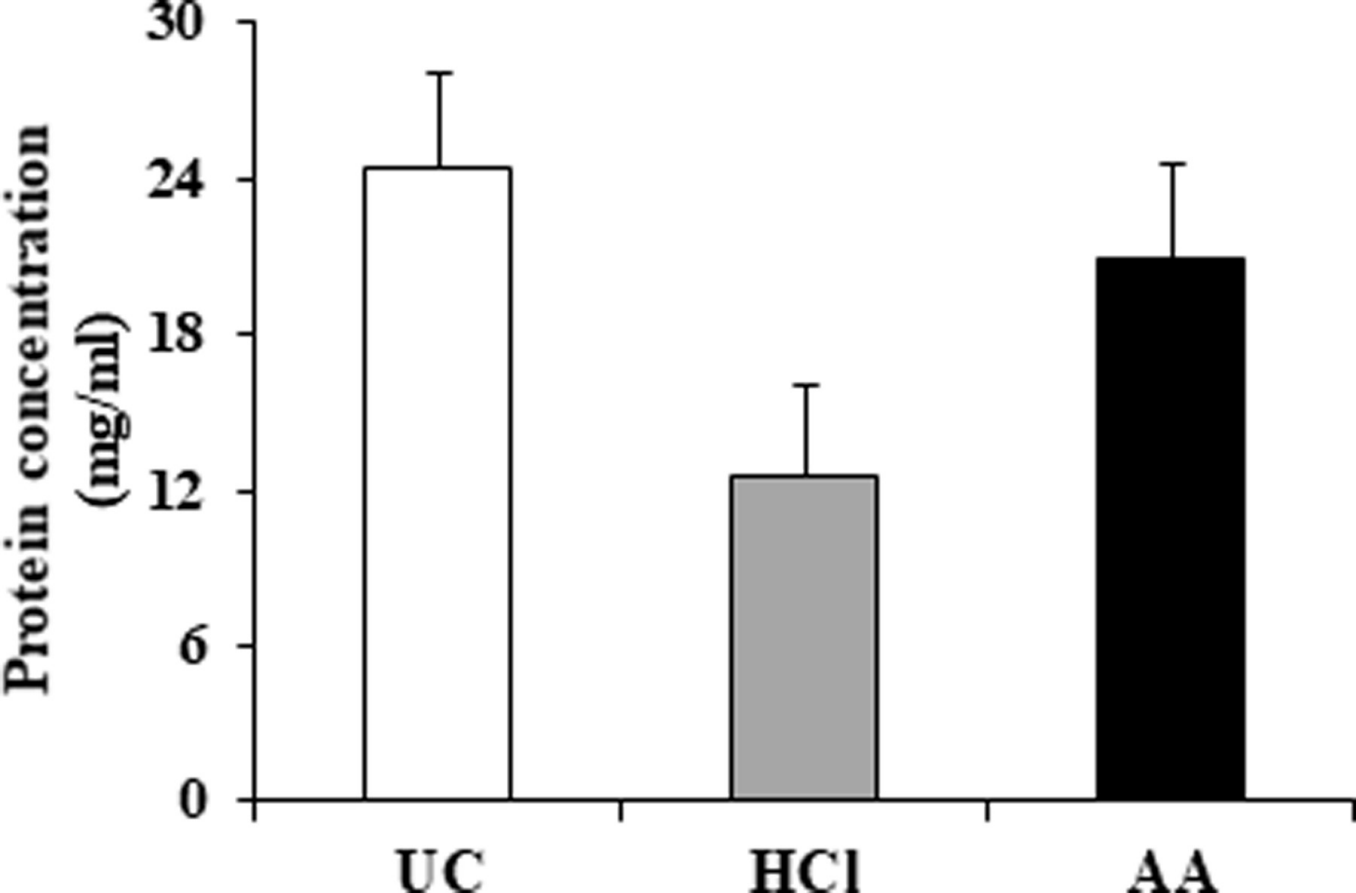
Protein concentration. Comparison of protein concentration in whey isolated by UC method, HCl-, or AA-treatments. Data from three independent experiments are represented as the mean ± standard deviation. Whey obtained by UC contained a higher protein concentration relative to that obtained by HCl- or AA-treatment (*p*>0.05).

Whey obtained by UC method contained a higher protein concentration (24.45±9.19 mg/mL) relative to that obtained by HCl- (12.47±0.96 mg/mL) or AA- (20.93±5.73 mg/mL) treatments (*p*> 0.05) (Fig 2).

### TRPS analysis

**Fig 3:**
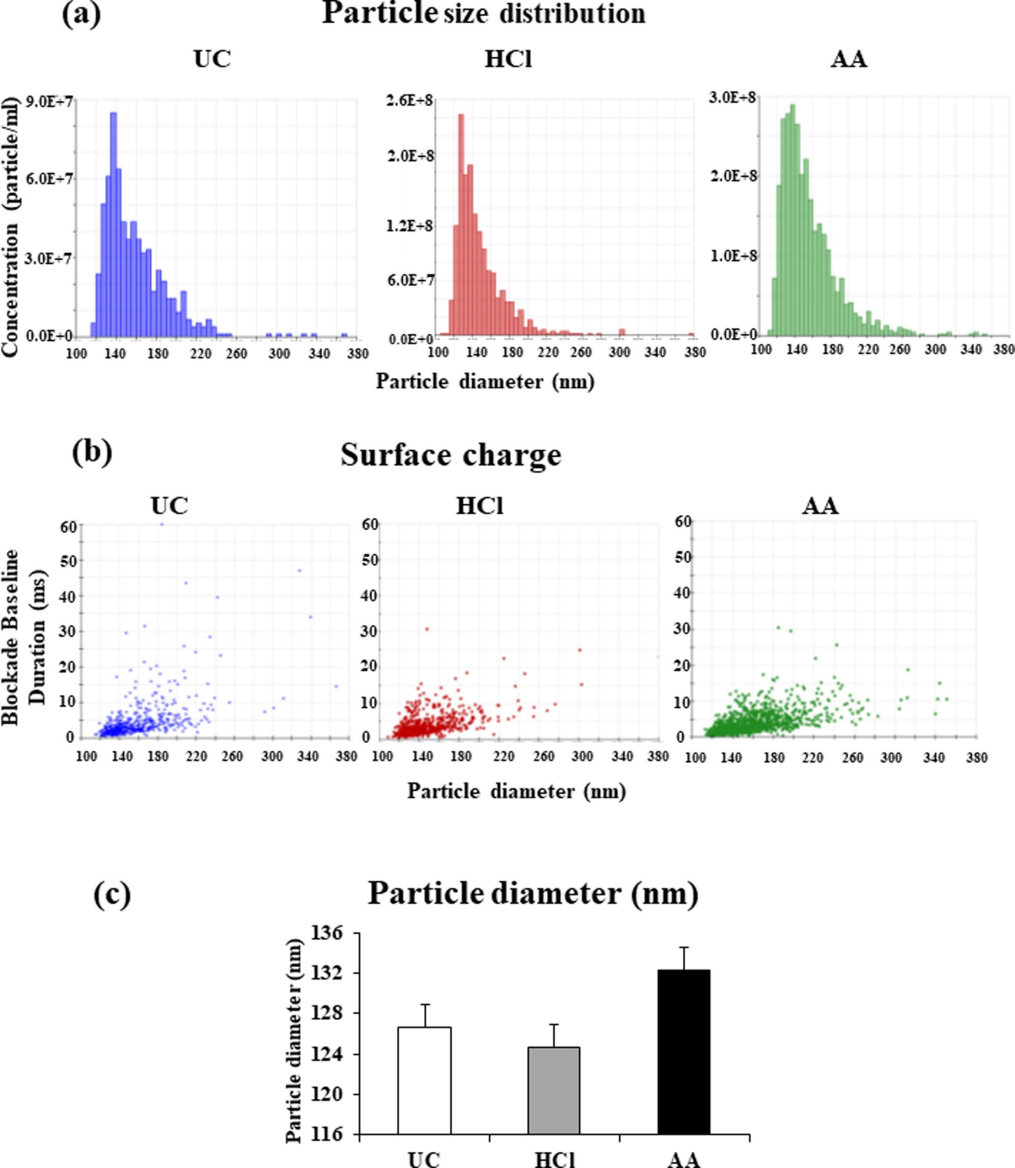
Particle size distribution and particle diameter. Comparison of the particle diameter of EVs in whey isolated by UC method, HCl-, or AA-treatments. Representative data from cow #1 were shown. (a) Particle size distribution and (b) surface charge were measured by TRPS. (c) Data from three independent experiments are represented as the mean ± standard deviation. Particle size did not differ significantly between the three methods (*p*>0.05).

**Fig 4:**
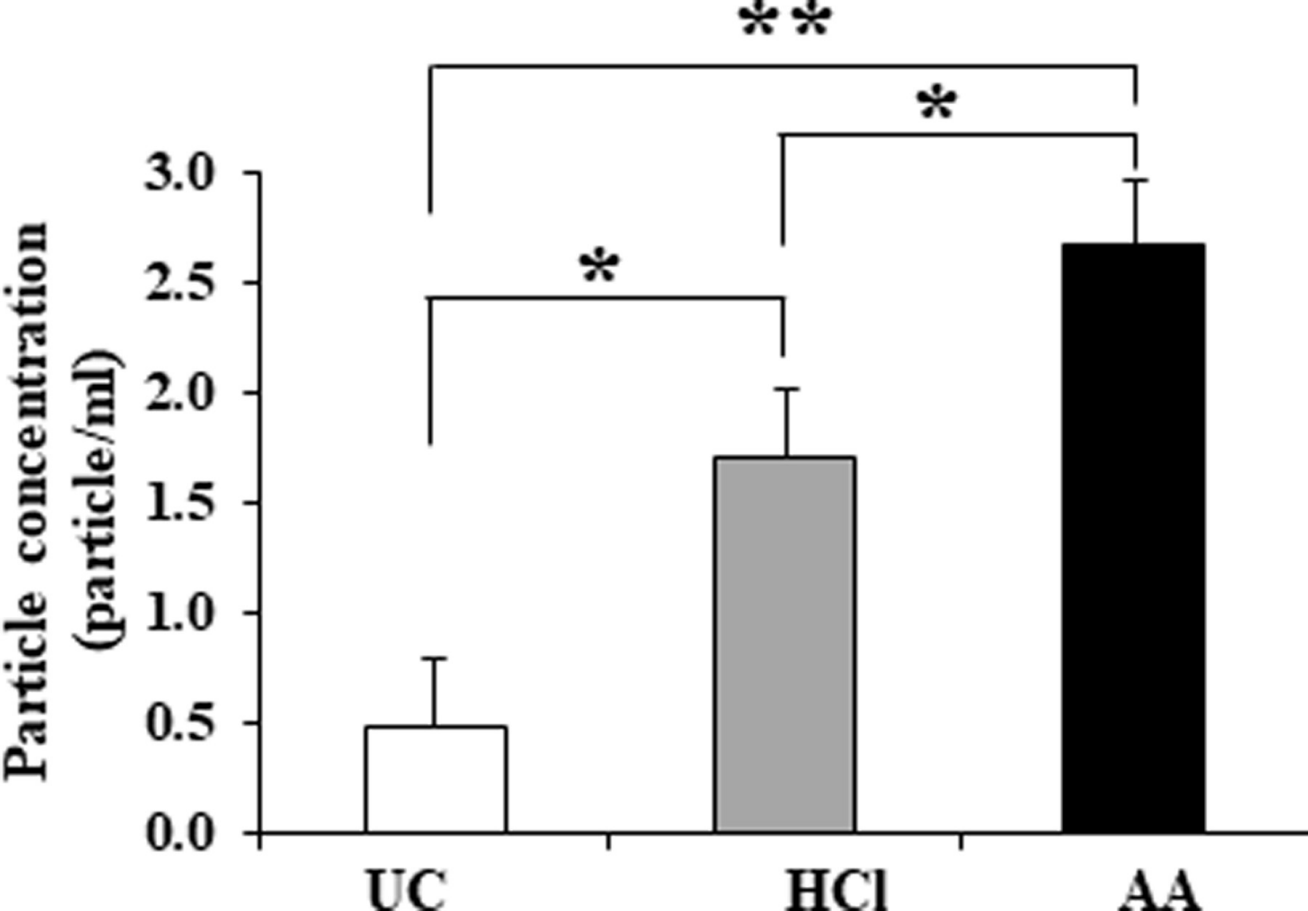
Particle concentration. Comparison of particle concentration in whey isolated by UC method, HCl-, or AA-treatments. Data from three independent experiments are represented as the mean ± standard deviation. The particle concentration of EVs differed significantly between the three methods. *p<0.05; **p<0.01.

TRPS analysis showed that the mean peak of particle diameter of EVs isolated by UC- or HCl- or AA-treatments were 126.66 nm, 124.66 nm, and 132.33 nm, respectively (Fig 3A and 3C). No significant difference in surface charge of EVs were observed among three methods (*p*> 0.05) (Fig 3B). Additionally, the particle concentration of EVs isolated by UC- or HCl- or AA-treatments were 4.8×10^8^ particles/mL, 1.7×10^9^ particles/mL, or 2.6×10^9^ particles/mL, respectively (Fig 4). These results suggested that the acid treatments yielded significantly higher amounts of EVs relative to UC (*p*<0.05).

### WB analysis

**Fig 5:**
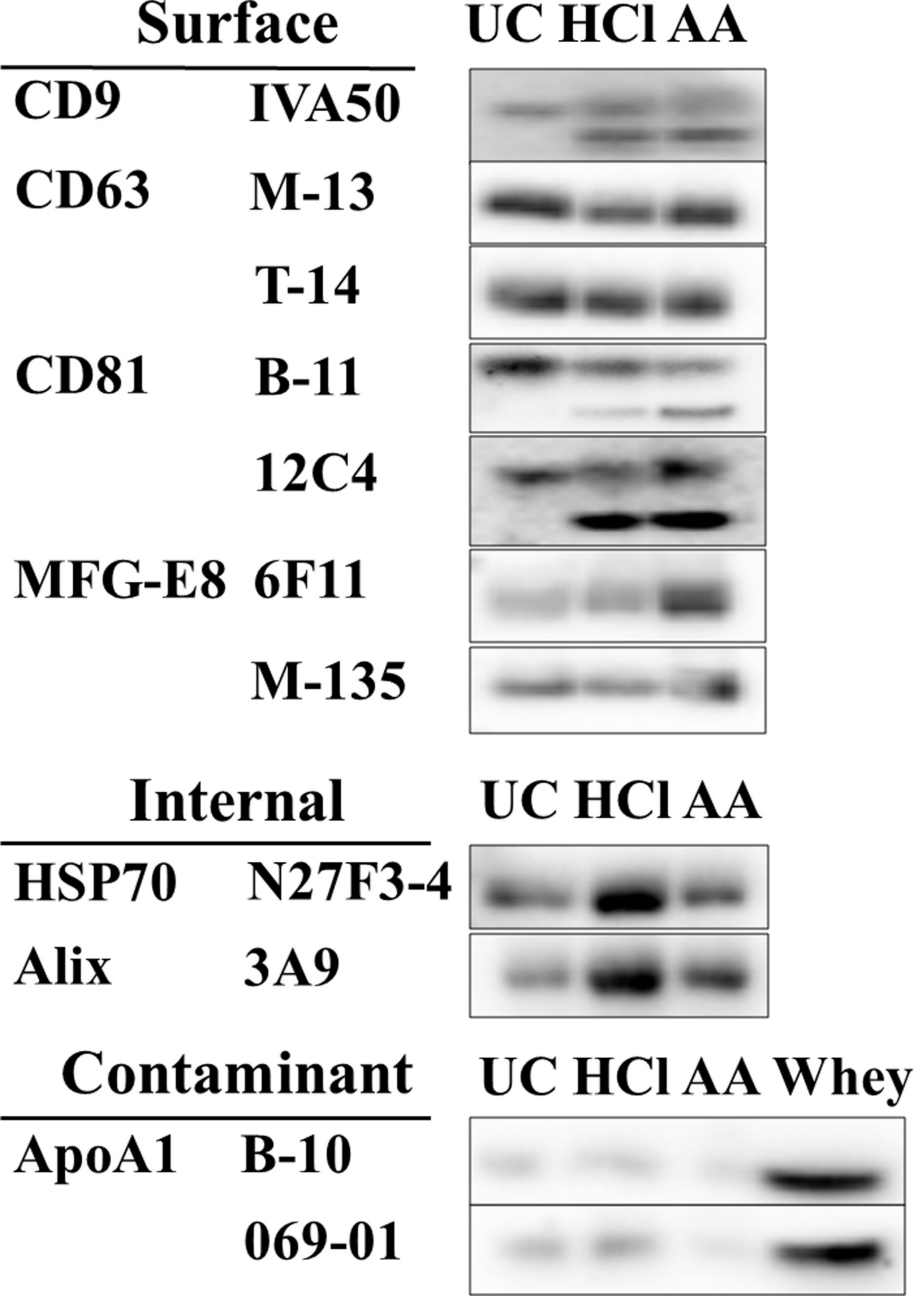
WB analysis using antibodies against EVs-marker proteins. WB analysis revealed partial degradation of the EV-surface-marker proteins CD9 and CD81 by acidification using HCl- or AA-treatment, whereas no change was observed in CD63, MFG-E8, HSP70, and Alix antibodies. ApoA1 antibodies were used for detection of contaminant using whey before purification of EVs.

WB analysis revealed detection of EVs-surface-marker proteins CD9, CD63, CD81, and MFG-E8 (Fig 5), with results using anti-CD9 and anti-CD81 showing bands at ≤24 kDa at similar intensities from samples treated with HCl and AA. This result indicated partial degradation of CD9 and CD81 on EVs by acidification, whereas no changes in the CD63, MFG-E8 surface markers were observed. Additionally, internal proteins of EVs were also detected by WB using HSP70 and Alix antibodies. Moreover, less amount of contaminant protein was observed in three methods by using ApoA1 antibodies.

## Discussion

In this study, we evaluated the effects of acidification on the isolation of milk EVs. Our results showed that morphology of EVs were spherical shape, similar to those of all isolates. However, rough surfaces were observed in some of the EVs isolated by HCl- or AA-treatment, which may be affected physically by acidification. Our results were consistence with a previous study [29]. Whey obtained by UC contained higher protein concentrations than that obtained by HCl- or AA-treatment. One explanation might be that non-EV proteins, including other debris, macromolecules, and protein aggregates, remained present in whey obtained by UC, which would be consistent with previous reports by Gheinani et al. [3] and Vaswani et al. [31]. Following HCl- or AA-treatment, the recovered whey contained relatively lesser protein but a higher amount of purified EVs than that obtained by UC. It is conceivable that isoelectric precipitation facilitated casein aggregation along with non-EV proteins and debris in order to promote efficient EV isolation. Moreover, acid treatment enhanced EV yield and purity relative to UC, and the particle concentration of EVs differed significantly between the three methods (*p*<0.05). Furthermore, our results agreed with previous studies demonstrating that low pH increased EV yield [27, 29]. However, although the EV-surface-marker proteins CD9, CD63, CD81, and MFG-E8 were detected by WB, CD9 and CD81 were partially degraded by acidification suggesting that the corrosive nature of the acids affected the outer structure of the EVs [29]. Whereas, the internal proteins of EVs were remain intact by acidification.

Our findings indicated that milk-derived EVs were affected by acidification, which effectively separated casein to yield highly pure EVs at increased levels. Milk is an abundant resource of EVs, and these findings suggest that acid treatment will be useful for isolation and large-scale production of EVs with reduced processing time relative to UC. Additionally, because milk EVs provide physiological [13] and pathological [19,20] information regarding the animals, the represent a promising source of potential biomarkers for diagnosing the progression of infectious diseases and/or the management of herd health. Moreover, milk EVs are resistant to stomach acid [32,33], making them potentially useful for clinical applications in humans for delivery of orally administered therapeutic agents passing through the gastrointestinal tract for entry into blood circulation.

## Conclusion

This study revealed that acidification was an easy and rapid approach for casein separation from milk promote efficient isolation and purification of EVs with reducing processing time, despite EV-surface proteins CD9 and CD81 were partially degraded. This study describes the acidification effects on EVs isolated from bovine milk for the first time. Our findings suggest that appropriate surface-marker antibodies should be used for accurate evaluation of EVs following isolation by acidification in downstream application.
